# Specific variants in *ftsI* reduce carbapenem susceptibility in *Pseudomonas aeruginosa*

**DOI:** 10.1128/spectrum.01027-25

**Published:** 2025-07-07

**Authors:** Hirokazu Yano, Noboru Nakata, Koji Yahara, Motoyuki Sugai, Yukari Sato

**Affiliations:** 1 Antimicrobial Resistance Research Center, National Institute of Infectious Diseases, Japan Institute for Health Security, Higashimurayama, Tokyo, Japan; 2 Leprosy Research Center, National Institute of Infectious Diseases, Japan Institute for Health Security 231182, Higashimurayama, Tokyo, Japan; University of Pittsburgh School of Medicine, Pittsburgh, Pennsylvania, USA

**Keywords:** penicillin-binding protein, PBP3, FtsI, carbapenem-resistant *Pseudomonas aeruginosa*, doripenem, meropenem, cefiderocol, CR-PA, resistance-associated point mutations

## Abstract

The *ftsI* locus of gram-negative bacteria encodes penicillin-binding protein 3 (PBP3), a common target of β-lactam antibiotics. Recent genomic surveillance of carbapenem-resistant *Pseudomonas aeruginosa* revealed that 17% of strains possessed nonsynonymous substitution in *ftsI*. However, the contribution of *ftsI* nonsynonymous substitution alone to the antimicrobial susceptibility of *P. aeruginosa* has not been formally evaluated. Therefore, we assessed the effect of *ftsI* nonsynonymous substitutions alone on β-lactam susceptibility in recombinant *P. aeruginosa* strains. The nonsynonymous substitutions C265G (Leu89Val), C1379T (Met460Thr), T1412G (Val471Gly), C1510T (Arg504Cys), C1579A (Pro527Thr), C1579T (Pro527Ser), C1599A (Phe533Leu), and G1609C (Val537Leu) were introduced into *ftsI* in an antimicrobial-susceptible strain via homologous recombination. Then, their β-lactam susceptibilities were investigated using the broth microdilution method and growth parameter analysis for the growth curves. The introduction of the N-terminal Leu89Val mutation did not alter β-lactam susceptibility. By contrast, the introduction of penicillin-binding (PB) domain mutations (Met460Thr, Val471Gly, Arg504Cys, Pro527Thr, Pro527Ser, Phe533Leu, or Val537Leu) reduced the susceptibility to meropenem and doripenem by two- to eightfold in MIC. Arg504Cys, the most common mutation encountered clinically, reduced susceptibility to cefepime by fourfold. Growth parameter analysis revealed that, conversely, these PB domain mutations generally increase the susceptibility to cefiderocol and piperacillin. The cocrystal structures of PBP3 with β-lactams suggest that the positions of PB domain mutations can affect the mobility of previously proposed key residues, namely, Tyr409, Arg489, Tyr503, Tyr532, Phe533, and the KSGT motif, via intramolecular interactions. These data demonstrated that seven specific variants in *ftsI* are genetic signals of bacterial adaptation to carbapenems.

**IMPORTANCE:**

Previous studies have reported the occurrence of *ftsI* mutations under various genetic backgrounds in *Pseudomonas aeruginosa* clinical isolates. However, the nature of *ftsI* mutations by themselves has not been thoroughly investigated under a common genetic background using recombinant strains. This study presents experimental data on the effect of *ftsI* point mutations alone on bacterial susceptibility to a wide range of antipseudomonal β-lactams. This study found that seven specific penicillin-binding domain mutations in FtsI/PBP3 reduced the efficacy of carbapenems but increased the efficacies of piperacillin and cefiderocol. This key finding will facilitate the development of therapeutic options for carbapenem-resistant *P. aeruginosa*-associated infections. Furthermore, this study contributes to the development of a reliable database collating resistance-associated point mutations, which would aid the interpretation of genomic data on *P. aeruginosa* clinical isolates.

## INTRODUCTION

Penicillin-binding proteins (PBPs) comprise a group of bacterial proteins involved in peptidoglycan synthesis, and they are targeted by *β*-lactam antibiotics. *Pseudomonas aeruginosa* carries eight PBPs: PBP1a (gene name: *ponA*, *locus_tag:* PA5045), PBP1b (*mrcB*, PA4700), PBP2 (*pbpA*, PA4003), *PBP3* (*ftsI*, also called *pbpB*, PA4418), PBP3x (*pbpC*, PA2272), PBP4 (*dacB*, PA3047), PBP5/6 (*dacC*, PA3999), and PBP7 (*pbpG*, PA0869) (1). Of these, PBP1a, PBP1b, PBP2, PBP3, and PBP3x are categorized as high-molecular-mass (HMM) PBPs. PBP1a and PBP1b possess transpeptidase and glycosyltransferase activities. PBP2, PBP3, and PBP3x only possess transpeptidase activity (2). The C-terminal regions of HMM PBPs constitute the penicillin-binding (PB) domain responsible for transpeptidase activity (2). Single-gene knockout of HMM PBPs negatively affects cellular fitness and influences cell shape, but only PBP3 is essential for bacterial growth (3). Other PBPs are categorized as low-molecular-mass (LMM) PBPs. LMM PBPs are considered nonessential according to observations in *Escherichia coli* (4). Among LMM PBPs, PBP5/6 in *P. aeruginosa* is considered a “decoy” target of β-lactams because of its efficient binding to β-lactams, nonessentiality, and high levels of production (1). In addition, PBP4 inactivation in *P. aeruginosa* induces AmpC overexpression, leading to high-level resistance to multiple β-lactams (5).

β-lactams exhibit PBP selectivity *in vitro* (6–8). For example, aztreonam, piperacillin, cefuroxime, cefotaxime, and ceftriaxone are selective for PBP3, whereas amoxicillin and cephalexin are selective for PBP4 in *E. coli*. Furthermore, β-lactams differ in their ability to penetrate the outer membrane. Specifically, carbapenems (imipenem, meropenem, and doripenem) penetrate the outer membrane more efficiently than aztreonam or piperacillin, leading to the rapid killing of *P. aeruginosa* (1). Thus, carbapenems should impose strong selective pressure on the PBP3-encoding gene *ftsI*.

A previous PCR-based survey on the *ftsI* locus of clinical *P. aeruginosa* isolates revealed the association of *ftsI* polymorphisms with β-lactam treatment history (9). In addition, whole-genome sequencing of *P. aeruginosa* isolates collected before and after ceftolozane–tazobactam treatment uncovered specific *ftsI* nonsynonymous substitutions (encoding Met460Thr or Arg504Cys mutations) in addition to other putative resistance mutations occurring during treatment (10). Similarly, *P. aeruginosa* isolates collected from a patient after meropenem treatment carried a nonsynonymous *ftsI* substitution (encoding the Arg504Cys mutation) and mutations in *mexR* and *oprD* (11). Furthermore, *P. aeruginosa* populations experimentally evolved in the presence of ceftazidime included clones carrying nonsynonymous substitutions (encoding Arg504Cys and Tyr503His mutations) in *ftsI* in addition to mutations in *ampC* and other loci (12).

Because the *ftsI*/PBP3 sequence is highly conserved in *P. aeruginosa*, it is hypothesized that most nonsynonymous substitutions occurring in *ftsI* in carbapenem-resistant *P. aeruginosa* (CR-PA) genomes are causal mutations for reduced susceptibility to β-lactams. However, clinical and experimentally evolved isolates carry additional mutations other than those in *ftsI* compared with the reference or ancestral strain. Thus, the exact trait conferred by the *ftsI* substitution alone remains unclear.

By inspecting publicly available genome sequences of CR-PA collected in two large surveillance studies (13, 14), we found that 17% of CR-PA isolates possess at least one nonsynonymous substitution in *ftsI*, comprising 41 nonsynonymous substitutions in 1,297 CR-PA genomes (Table S1). Per previous cocrystal structure analysis, β-lactams interact with overlapping but different sets of amino acid residues in PBP3 (15). Therefore, the PBP3 substitutions might exert different effects on bacterial susceptibility among different β-lactams. In this study, we hypothesized that *ftsI* nonsynonymous mutations found in CR-PA isolates are causal mutations for reduced susceptibility to specific β-lactam antibiotics. To this end, we introduced eight nonsynonymous substitutions found in clinical strains into the *ftsI* locus in the chromosome of an antibiotic-susceptible strain via homologous recombination, and we then determined the susceptibility profiles of the recombinant strains.

## RESULTS

### Nonsynonymous substitutions in *ftsI* in meropenem-resistant *P. aeruginosa*

To identify clinically relevant nonsynonymous substitutions, a global genomic data set of 1,297 modern CR-PA isolates collected from 2018 to 2020 based on the criterion of meropenem MIC ≥8 was constructed (see “Materials and Methods” for details). In the *ftsI* alignment, 41 nonsynonymous substitutions occurring in 38 codons were identified. Twelve substitutions occurred in the PBP3 N-terminal domain-coding region (amino acids 1–200), which includes the N-terminal subdomain and head subdomain (16), and 29 substitutions occurred in the PB domain-coding region (amino acids 201–579; Table S1). Substitutions discussed here are summarized in Table 1. In total, 9.2% (119/1,297) of CR-PA isolates carried nonsynonymous substitutions in the N-terminal domain-coding region, whereas 9.8% (127/1,297) of isolates featured nonsynonymous substitutions in the PB domain-coding region. Twenty-two substitutions were detected in only one isolate (variant allele frequency of 0.08%). Among the remaining 19 types, eight substitutions (encoding Thr91Ala, Asn117Ser, Met460Thr, Val471Gly, Arg504Cys, Pro527Ser, Phe533Leu, and Val537Leu mutations) were detected in at least two Japanese isolates collected in a recent national CR-PA surveillance (14) (Table 1), and the nature of these substitutions was investigated. Relatively common substitutions included A271G (encoding Thr91Ala; allele frequency of 5.0%), C1510T (Arg504Cys, 3.1%), C1599A (Phe533Leu, 2.7%), and A350G (Asn117Ser, 2.6%), and the remaining substitutions were extremely rare (<1%) in CR-PA. Among nonsynonymous substitutions uncommon in Japanese isolates, T13C (Tyr5His, allele frequency <1%), C644T (Pro215Leu, <1%), C718G (Leu240Val, <1%), and GCC (415–417) to AGT or TCC (both encoding Ala139Ser, 1%) were detected in more than one CR-PA isolate (Table 1).

**TABLE 1:** *ftsl* nonsynonymous substitutions discussed in this study*^b^*^,*c*^

Nonsynonymous substitution^*a*^	Variant allele frequency in 1,297 genomes (fraction)	Amino acid substitution	Location
T13C	11 (0.0085)	Tyr5His	N-term
**C265G**	3 (0.0023)	**Leu89Val**	N-term
A271G	66 (0.0508)	Thr91Ala	N-term
A350G	34 (0.0262)	Asn117Ser	N-term
GCC (415–417) to TCC or AGT	13 (0.010)	Ala139Ser	N-term
C644T	2 (0.0015)	Pro215Leu	PB
C718G	10 (0.0077)	Leu240Val	PB
**C1379T**	2 (0.0015)	**Met460Thr**	PB
**T1412G**	8 (0.0062)	**Val471Gly**	PB
G1420T	2 (0.0015)	Val474Ser	PB
**C1510T**	40 (0.0308)	**Arg504Cys**	PB
**C1579A**	1 (0.0008)	**Pro527Thr**	PB
**C1579T**	4 (0.0031)	**Pro527Ser**	PB
**C1599A**	35 (0.0270)	**Phe533Leu**	PB
**G1609C**	8 (0.0062)	**Val537Leu**	PB

^*a*^ All nonsynonymous substitutions are listed in Table S1 of supplemental material. Mutations characterized in this study are shown in bold.

^*b*^ N-term.

^*c*^ N-terminal domain.

Cloning and allelic exchange experiments were designed to examine the effects of these variants on β-lactam susceptibility in a common genetic background. However, the nonsynonymous substitutions A271G (Thr91Ala) and A350G (Asn117Ser) in the N-terminal domain could not be introduced into the cloned *ftsI* in *E. coli*. The position of another nonsynonymous substitution, namely C265G encoding the Leu89Val mutation, was close to A271, and the substitution was successfully introduced into the cloned *ftsI*. Thus, the effect of the C265G substitution was investigated as a representative mutation in the N-terminal domain. Although the C1579T mutation (encoding Pro527Thr) was detected in only one isolate, the substitution of the same site (C1579T encoding Pro527Ser) was detected in four isolates. Thus, Pro527Thr was also investigated as a candidate resistance mutation.

**Fig 1:**
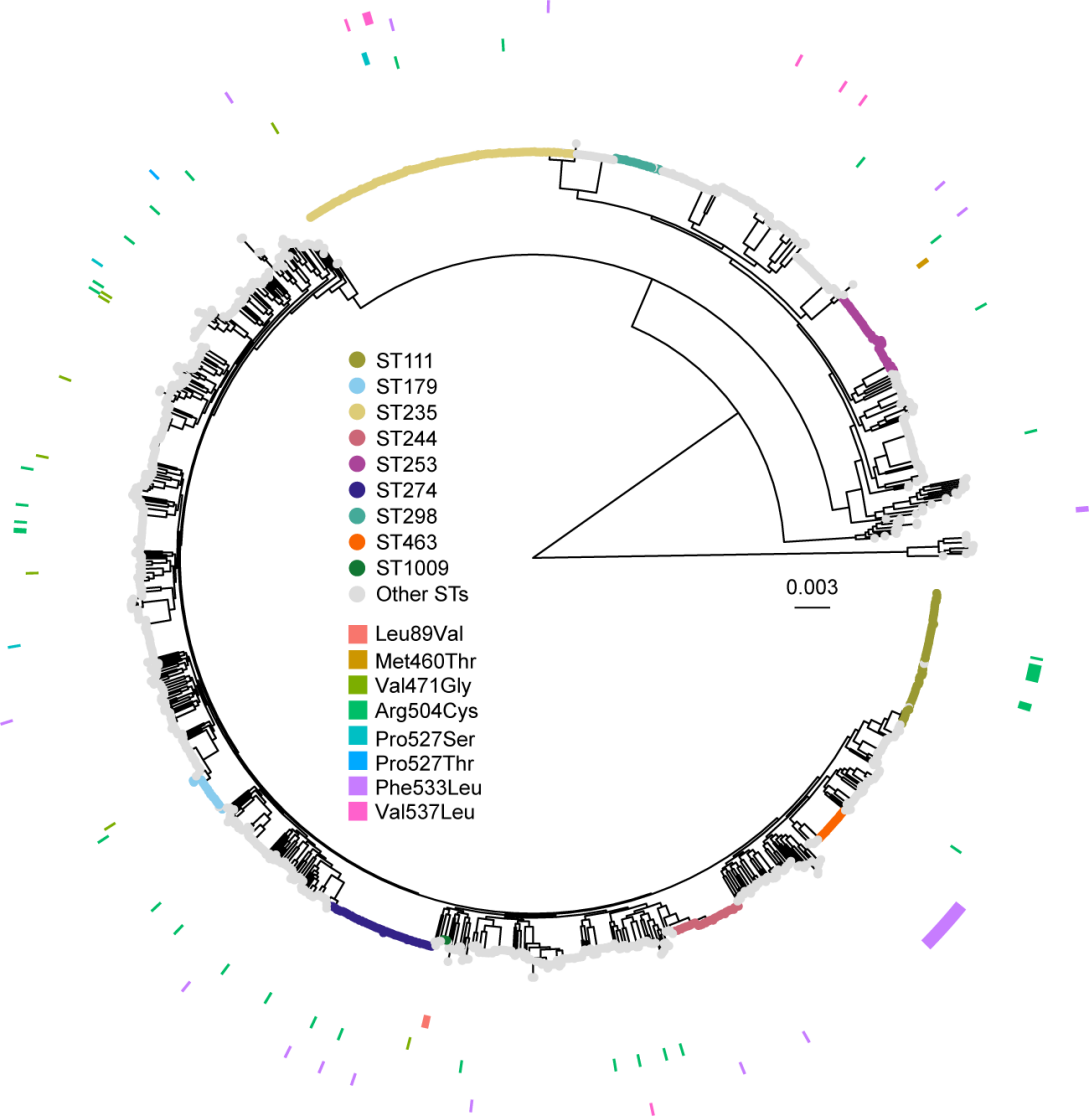
Distribution of FtsI/PBP3 mutations in 1,297 CR-PA isolates. The data set originated from two recent studies on meropenem-resistant isolates (13, 14). The diagram in the center is a maximum likelihood phylogenetic tree based on core gene alignments. The tip color represents STs. Outer rings represent the presence/absence pattern of FtsI/PBP3 mutations: from center to outer, Leu89Val, Met460Thr, Arg504Cys, Pro527Ser, Pro527Thr, Phe533Leu, and Val537Leu. The countries where the strains were isolated are listed in Table S1.

In summary, eight nonsynonymous substitutions encoding Leu89Val, Met460Thr, Val471Gly, Arg504Cys, Pro527Ser, Pro527Thr, Phe533Leu, and Val537Leu mutations were individually introduced into the cloned *ftsI* in *E. coli*. Among these, Leu89Val, Met460Thr, and Pro527Thr mutations were detected only in Japanese isolates. These PBP3 mutations were detected only in the members of sequence type (ST) ST1009, ST253, and ST4123, respectively (Fig. 1). The other five substitutions were present in isolates from at least one other country and were distributed in multiple STs, including global high-risk clones such as ST235, ST244, and ST111 (17). Notably, the substitution encoding the Phe533Leu mutation is fixed in ST463, which has been exclusively isolated in China (Fig. 1) (13).

Changes in cellular viability attributable to PBP3 inactivation upon β-lactam binding were normally masked by the influx efficiency of β-lactams and expression of PDC β-lactamase and RND family efflux pumps (18, 19). To detect a more direct impact of PBP3 inactivation on cellular viability, we sought to eliminate the influence of efflux pumps on the experiments. Therefore, we introduced the *ftsI* substitutions into the chromosome of drug-susceptible strain YM64, in which four *mex* operons were deleted from the chromosome (20) (see “Materials and Methods”).

### Susceptibility profiles of recombinant strains

The MICs of β-lactams against the eight recombinant strains were determined using the broth microdilution method. For the susceptibility test, eight antimicrobials were selected to cover five antipseudomonal classes: penicillin, cephem, carbapenem, monobactam, and cefiderocol (Table 2). The MICs of the recombinant strain carrying the Leu89Val mutation were identical to those of the parent strain. By contrast, the seven other recombinant strains carrying PBP3 PB domain mutations exhibited an increased doripenem MIC. These strains also exhibited a higher meropenem MIC, but the increase was greater for doripenem (four- to eightfold) than for meropenem (two- to eightfold; Table 2). The seven PBP3 PB domain mutations commonly increased imipenem-relebactam MIC by twofold; however, this increase was not observed in imipenem MIC for Met460Thr, Pro527Thr, and Phe533Leu. Exceptionally, the strain with the most clinically common mutation, Arg504Cys, exhibited fourfold higher cefepime MIC. Although the effect is minor, strains carrying the Pro527Thr and Pro527Ser variants also became less sensitive to cefepime. Furthermore, Arg504Cys increased ceftolozan-tazobactam MIC by twofold. None of the recombinant strains exhibited increased MICs for piperacillin or aztreonam. Reduced piperacillin MIC was observed for Val471Gly, Pro527Thr, Pro527Ser, Phe533Leu, and Val537Leu mutations. Notably, all seven strains carrying PB domain mutations displayed a lower cefiderocol MIC than the two strains with the wild-type (WT) PB domain.

**TABLE 2:** β-lactam MICs against *P. aeruginosa* YM64 derivative strains producing FtsI/PBP3 variant

Strain	FtsI/PBP3 mutation	MIC (μg/mL)*^a^*									
		PIP	CAZ	FEP	C/T	IMP	IMP/REL	MEM	DRP	ATM	FDC
YM64	WT	0.5	0.5	0.125	0.25	1	0.25	0.25	0.5	0.25	0.063
PAY7	Leu89Val	0.5	0.5	0.125	0.25	1	0.25	0.25	0.5	0.25	0.063
PAY53	Met460Thr	0.5	0.5	0.125	0.25	1	0.5	0.5	**2**	0.125	≤0.031
PAY49	Val471Gly	0.25	0.5	0.125	0.25	2	0.5	**1**	**2**	0.25	≤0.031
PAY47	Arg504Cys	0.5	1	**0.5**	0.5	2	0.5	**1**	**4**	0.25	≤0.031
PAY18	Pro527Thr	0.25	0.5	0.25	0.25	1	0.5	**1**	**2**	0.25	≤0.031
PAY45	Pro527Ser	0.25	0.5	0.25	0.25	2	0.5	0.5	**2**	0.25	≤0.031
PAY42	Phe533Leu	**0.125**	0.5	0.125	0.25	1	0.5	**2**	**4**	0.125	≤0.031
PAY51	Val537Leu	0.25	0.5	0.125	0.25	2	0.5	**1**	**4**	0.25	≤0.031

^*a*^ Abbreviations used are as follows: PIP, piperacillin; CAZ, ceftazidime; FEP, cefepime; C/T, ceftolozan-tazobactam; MEM, meropenem; IMP, imipenem; IMP/REL, imipenem-relebactam; DRP, doripenem; ATM, aztreonam; FDC, cefiderocol. The values represent the median of three or five independent experiments performed on different days, excluding ceftolozane-tazobactam and imipenem-relebactam experiments, for which the experiment was performed twice and identical values were obtained. The values equal to or higher than fourfold increased/decreased MIC were shown in bold.

### Effect of PBP3 mutations on growth parameters

**Fig 2:**
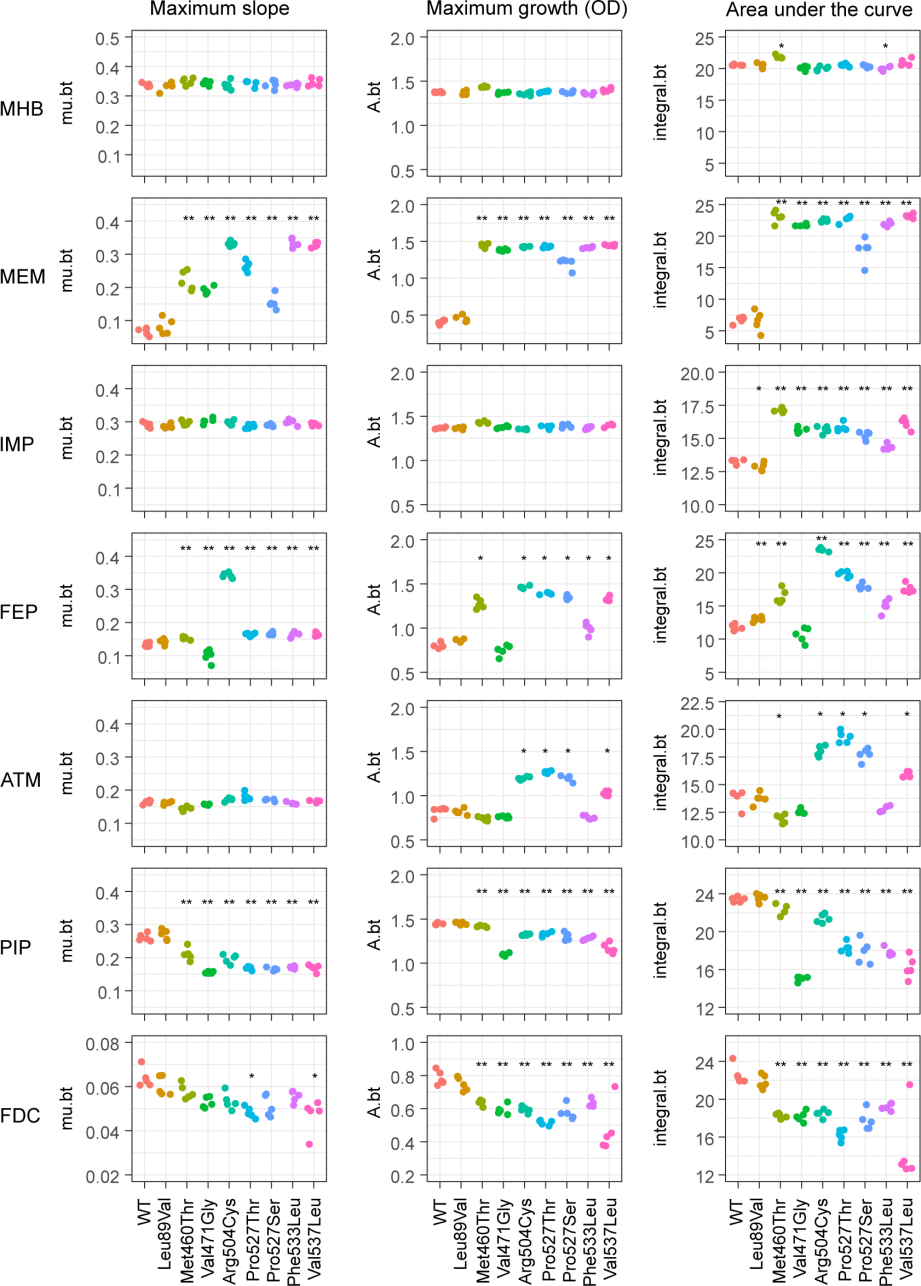
Effect of FtsI/PBP3 mutations on growth parameters. Left, maximum slope. Middle, maximum OD of the growth curve. Right, AUC. Growth curves are provided in Fig. S1. Statistical significance for the difference between the parent strain (WT) and strains with PBP3 mutations was evaluated by a two-sided Wilcoxon rank sum test, followed by *P*-value adjustment by the Benjamini-Hochberg method: *, adjusted *P*-value < 0.05; **, adjusted *P*-value < 0.01. Medium conditions used were as follows (from top row to bottom row): cation-adjusted MHB; MHB containing meropenem 0.125 µg/mL; imipenem 1 µg/mL; cefepime 0.063 µg/mL; aztreonam 0.125 µg/mL; piperacillin 0.063 µg/mL; and iron-depleted MHB containing cefiderocol 0.002 µg/mL.

Regarding sensitivity, the broth microdilution method used in the susceptibility test uses a discrete variable as the MIC. Therefore, we conducted a more sensitive growth parameter analysis for the growth curves to capture minor fitness effects of PBP3 mutations in the presence of antimicrobials. The growth of test strains in microtiter plates was monitored for 24 h for experiments involving meropenem, imipenem, piperacillin, cefepime, and aztreonam and for 48 h for experiments involving cefiderocol. Next, the maximum slope (mu.bt of grofit output), maximum OD (A.bt), and area under the curve (AUC; integral.bt) of growth curves were obtained for five replicate cultures (Fig. 2). The OD plots are provided in Fig. S1. In cation-adjusted Mueller-Hinton broth (MHB), no difference was observed in either maximum slope or maximum OD between the parent strain and the recombinant strains producing PBP3 variants. Concerning AUC, an increase (6% of the wild-type mean) was observed for Met460Thr, whereas a slight decrease (3%) was observed for Phe533Leu. Thus, almost no detectable fitness cost arose from the PB domain mutations in MHB (Fig. 2). In the presence of meropenem (0.125 µg/mL), the maximum slope, maximum OD, and AUC were significantly increased for the seven strains with PB domain mutations; an increase in AUC was 160%–230% of the wild-type mean, confirming the positive fitness effects of these mutations (adjusted *P*-value < 0.01 in the two-sided Wilcoxon rank sum test) in the presence of meropenem. Moreover, with imipenem (1 µg/mL), the maximum slope and maximum OD did not differ among the strains. However, the AUCs of the seven strains with PB domain mutation were significantly higher than that of the parent strain with wild-type PBP3 (an increase was 8%–19%, adjusted *P*-value < 0.01). This was primarily attributable to the shortened lag phase in strains with PB domain mutations (Fig. S1). This shortened lag phase in strains with PB domain mutations was barely observed when the imipenem concentration was decreased to 1/2 MIC (0.5 µg/mL); instead, an increase in the maximum slope (4%–11%) was observed for strains carrying Met460Thr, Val471Gly, Pro527Thr, and Pro527Ser (Fig. S1). Therefore, although the imipenem MIC did not increase for three PB domain mutations, the positive fitness effect of all PB domain mutations was detected at the growth parameter level, and the positive fitness effect of the seven PB domain mutations was common across carbapenems.

MIC measurements indicated the positive fitness effects of Arg504Cys, Pro527Thr, and Pro527Ser mutations in the presence of cefepime. In the growth curves, the positive fitness effects of Met460Thr, Val537Leu, and the above three mutations were detected as an increase in all three growth parameters (Fig. 2). Notably, Arg504Cys increased the maximum slope by 157%. Among PB domain mutations, Val471Gly exceptionally displayed a decreased maximum slope in the presence of cefepime; however, it did not significantly affect the AUC. Although the aztreonam MIC did not increase for strains harboring PBP3 variants, the positive fitness effect of four PB domain mutations (Arg504Cys, Pro527Thr, Pro527Ser, and Val537Leu) was detected as a 25%–54% increase in the maximum OD, resulting in increasing AUCs by 10%–35% (Fig. 2 and S1). The negative fitness effects of Met460Thr and Phe533Leu in the presence of aztreonam were suggested by the MICs. In the growth curves, the AUC decreased by 15% for Met460Thr and 12% for Phe533Leu.

The decrease in the piperacillin MIC for five strains harboring PB domain mutations (Val471Gly, Pro527Thr, Pro527Ser, Phe533Leu, and Val537Leu) was reflected as significant decreases in all three growth parameters in the presence of a low concentration (1/8 MIC) of piperacillin (adjusted *P*-value < 0.01; Fig. 2 and S1). Although we could not detect the effect of Met460Thr and Arg504Cys on the piperacillin MIC, their weakly negative effects on growth were detected as statistically significant decreases in all three growth parameters (Fig. 2). The decrease in the AUC ranged from 5% for Arg504Cys to 36% for Val471Gly.

Cefiderocol susceptibility tests were performed using iron-depleted MHB, which is suboptimal for bacterial growth. The six strains harboring PB domain mutations exhibited a slightly decreased maximum slope in iron-depleted MHB without cefiderocol, whereas the maximum OD did not differ among the strains, resulting in a 3%–6% decrease in the AUC for the Pro527Thr, Phe533Leu, and Val537Leu mutations (Fig. S1). In the presence of cefiderocol, a decrease in the maximum slope was detected for only two strains harboring PB domain mutations. However, the maximum OD and AUC were significantly decreased for all seven strains harboring PB domain mutations (adjusted *P*-value < 0.01). Concerning the AUC, the extent of the decrease ranged from 15% for Phe533Leu to 35% for Val537Leu. A significant decrease in the AUC was associated with an extended lag phase and decreased maximum OD for all PB domain mutants (Fig. S1). These cefiderocol medium-specific effects of PB domain mutations on growth were consistent with the observation in MIC measurements.

Altogether, the growth parameters illustrated that the PB domain mutations typically increase cefiderocol and piperacillin susceptibilities in exchange for reducing carbapenem susceptibility, highlighting a trade-off associated with PBP3 evolution occurring in the clinical setting.

### Molecular interactions around the PB domain mutation positions

**Fig 3:**
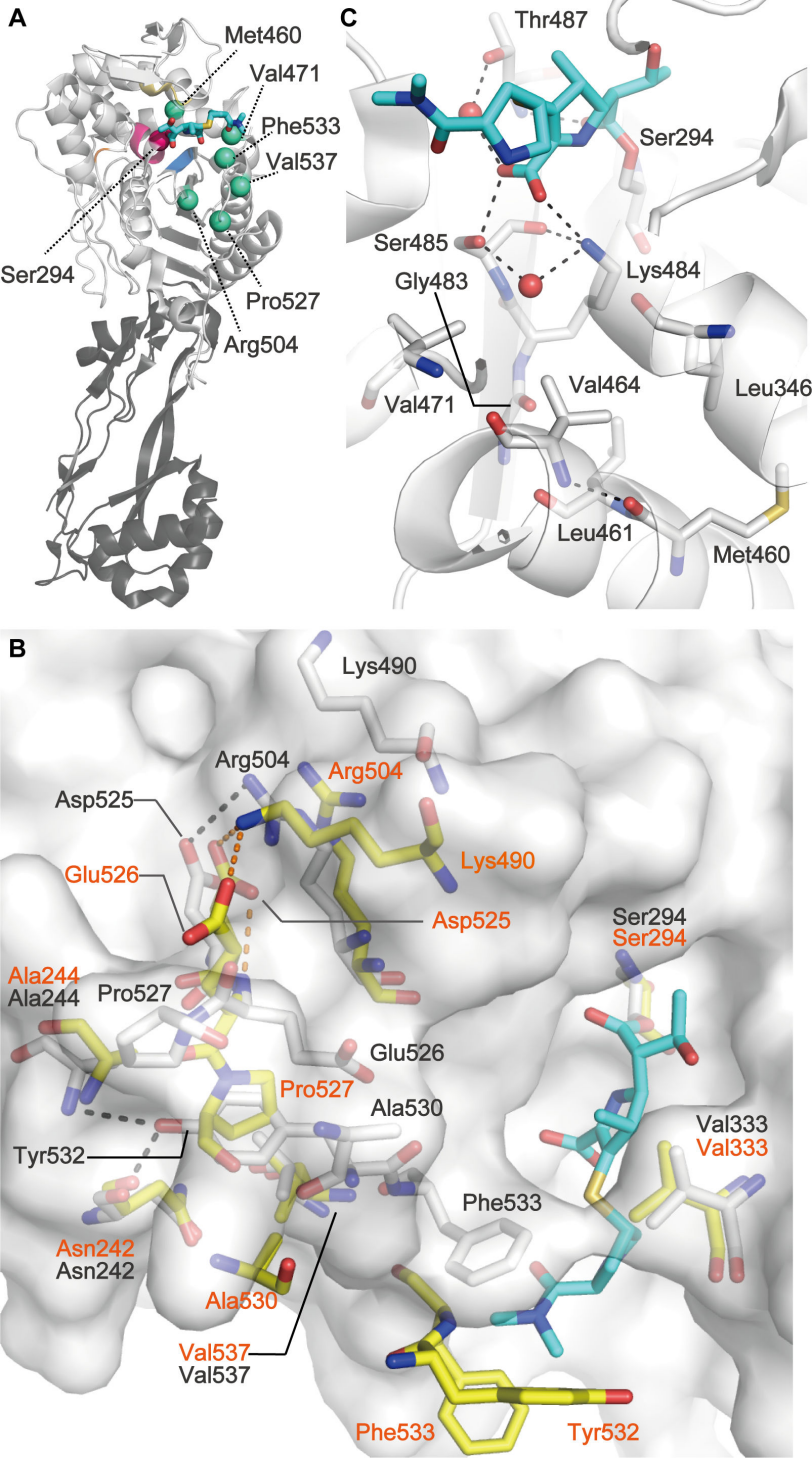
Molecular interactions around the active site region of PBP3. The stick model in cyan is meropenem. (**A**) Overall structure of PBP3 with meropenem bound to Ser294 (magenta). Data originate from PDB code: 3PBR. Dark gray, N-terminal domain. Light gray, PB domain. PB domain mutation positions identified as affecting β-lactam susceptibility are highlighted in green spheres. The PBP3 active site comprises nine residues: S and K of SXXK motif (magenta), S and N of SXN motif (yellow), KSGT motif (blue), and Gly410 (orange). (**B**) Meropenem-induced conformational change in PBP3. The molecular surface of PBP3 in the complex is shown in gray. The PBP3-meropenem complex (white) was superposed onto the apo structure (yellow) of PBP3; RMSD = 1.1 Å for 429 Cα atoms. Black dashed lines indicate hydrogen bonds and a salt bridge in the complex. The yellow dashed line indicates a hydrogen bond in the apo structure. Data originate from PDB codes 3PBR and 3PBN. (**C**) Val471 and Met460 near the KSGT motif in the complex. Dashes, hydrogen bonds; red sphere, water molecule. Amino acid labels of the PBP3-meropenem complex and the apo structure are shown in black and orange, respectively.

A previous study (15) determined the crystal structures of *P. aeruginosa* PBP3 bound by various substrates, including meropenem (PDB code: 3PBR) and ceftazidime (PDB code: 3PBO), as well as its apo form (PDB code: 3PBN). The PBP3 active site encompasses nine residues broadly conserved in PBPs: the active serine (Ser294) followed by lysine at the beginning of the α2 helix, forming the SXXK motif (residues 294–297); a second motif SXN (residues 349–351) in a loop between the α4 and α5 helices; the third motif consisting of the residues Lys-A-Gly-A: where A is serine or threonine (KSGT motif: residues 484–487); and the ninth residue, namely glycine at position 410 (Gly410) in the rear of the active site (Fig. 3A) (2, 15). Han et al. (15) reported a ligand-introduced conformational rearrangement of the flexible loop connecting the β5 sheet and α11 helix (residues 526–533) and a ligand-introduced conformational change of Tyr409, Arg489, and Tyr503 in the wild-type PBP3 of *P. aeruginosa*. Therefore, we revisited the publicly available crystal structures of the wild-type PBP3 and investigated the presence of intramolecular interactions involving the PB domain mutation positions influencing substrate binding.

#### Phe533Leu

Phe533 is a part of the flexible loop. Phe533 is the second most common mutation position in the PB domain of CR-PA PBP3. The MICs of carbapenems possessing a pyrrolidine at the C2 position in meropenem and doripenem were increased by the Phe533Leu mutation. Phe533 is reported to form a tunnel-like hydrophobic pillar with Val333 to stabilize the pyrrolidine at the C2 position in the PBP3-meropenem complex (Fig. 3B) (16). Substitution of the bulky Phe533 with leucine might reduce the stabilization effect of the residue on the pyrrolidine at the C2 position of meropenem (15).

#### Val537Leu

Val537 forms hydrophobic interactions with Phe533 and Tyr532, whereas it forms van der Waals interactions with Asn242 in the PBP3-meropenem complex (Fig. 3B white sticks). Tyr532 flipping, assisted by Asn242 and Asn242, occurs jointly with hydrophobic pillar formation by Phe533 and Val333 (Fig. 3B). Substitution of Val537 with the bulkier residue leucine makes the residue closer to Tyr532 and Asn242, which might increase their interactions (Fig. S2). This could affect Tyr532 flipping and the flexible loop structure, consequently reducing the stabilization effect of the hydrophobic pillar on meropenem.

#### Pro527Thr and Pro527Ser

Pro527 is a part of the flexible loop. In the apo structure, Pro527 forms hydrophobic interactions with Ala530 (Fig. 3B yellow sticks). When meropenem binds to PBP3, a conformational change is introduced into the flexible loop, preventing Pro527 from interacting with Ala530. Consequently, Pro527 forms van der Waals interactions with Tyr532 (Fig. 3B). Thus, the replacement of Pro527 with threonine or serine might weaken the residue’s influence on Tyr532 and then reduce the stabilization effect of the hydrophobic pillar on meropenem.

#### Met460Thr

Met460 has a hydrophobic interaction with Leu346 and a hydrogen bond with the main chain amide of Val464 in the PBP3-meropenem complex (Fig. 3C). Leu346, Val464, and Val471 form a hydrophobic patch and interact with Lys484, Gly483, and Ser485, respectively, two of which comprise the KSGT motif, on the β3 sheet (Fig. 3C). The C3 carboxylic acid group of meropenem forms a hydrogen bond with Lys484. Furthermore, Leu461 adjacent to Met460 interacts with Lys484 and Gly483. Thus, the replacement of Met460 with threonine might reduce the interactions between Lys484 and the C3 carboxylic acid group of meropenem via Leu346, Leu461, Val464, and Gly483.

#### Val471Gly

The Val471Gly mutation was previously observed in the genomes of experimentally evolved P14 derivative clones in the presence of doripenem (21). The Val471 side chain forms van der Waals interactions with the Ser485 side chain of the KSGT motif (Fig. 3C). The Ser485 main chain forms a hydrogen bond with the C3 carboxylic acid group of meropenem in the complex. Substitution of Val471 with the smaller amino acid glycine might abrogate the interaction of the residue with Ser485. Consequently, the interactions between Ser485 and meropenem might be reduced.

#### Arg504Cys

Arg504 is the most common mutation in the PB domain of CR-PA PBP3. In the apo structure, Arg504 does not interact with Asp525. The Asp525 side chain forms a salt bridge with the Lys490 side chain, which forms a salt bridge with the side chain of Glu526 (Fig. 3B). The Asp525 side chain also forms hydrogen bonds with the main chain amide of Glu526 (Fig. 3B). When meropenem binds to PBP3, Arg504 forms a salt bridge with the side Asp525 chain, and Asp525 abrogates interactions with Lys490 and Glu526. Lys490 also abrogates interaction with Glu526. These conformation changes introduce van der Waals interactions between Glu526 and Tyr532. Thus, the substitution of Arg504 with cysteine might mitigate the influence of Asp525 to Tyr532, thereby reducing the stabilization effect of the hydrophobic pillar on meropenem.

**Fig 4:**
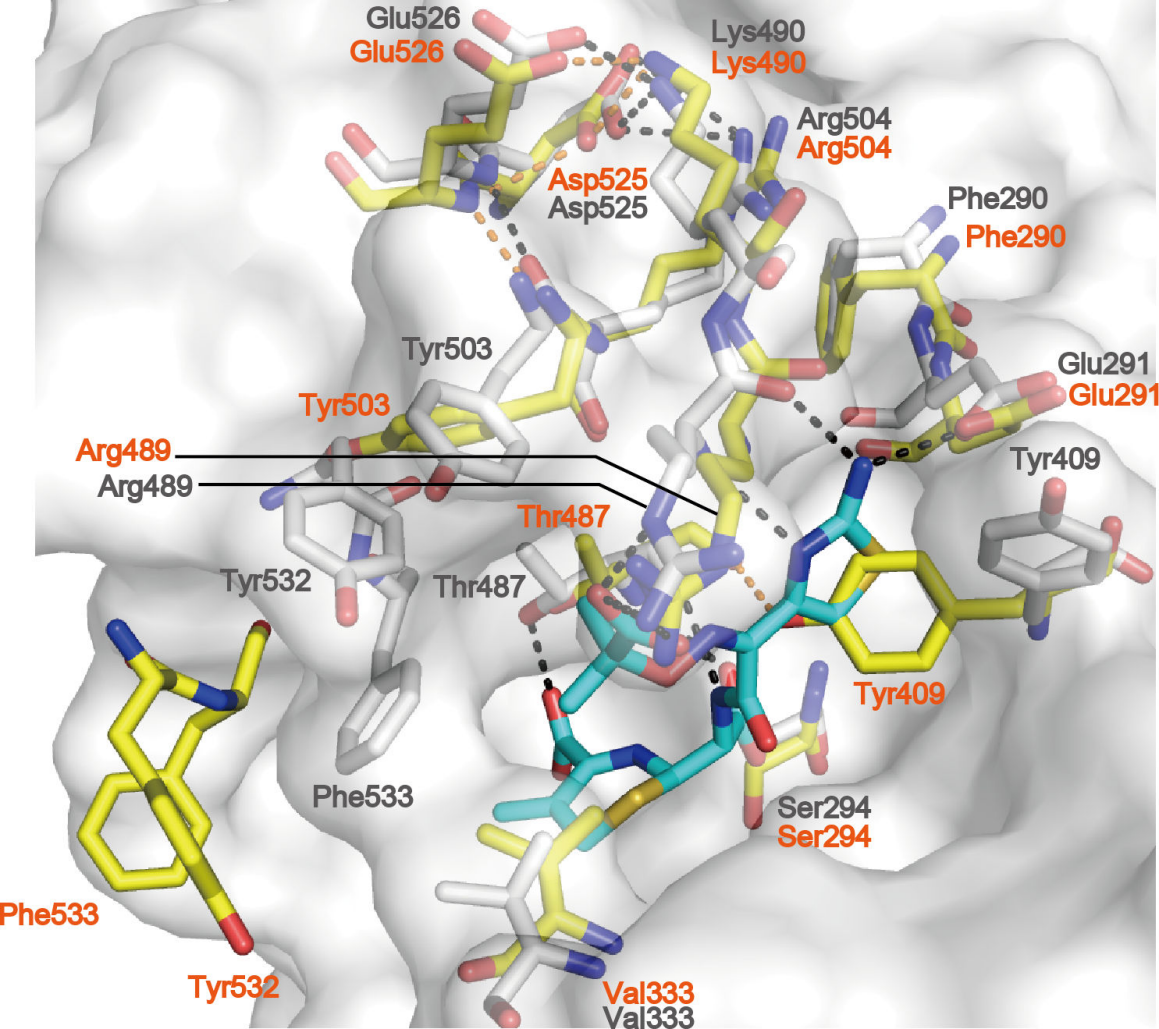
Ceftazidime-induced conformational change in the active site region of PBP3. The PBP3-ceftazidime complex (white) was superposed onto the apo structure (yellow); RMSD = 1.0 Å for 430 Cα atoms. The molecular surface of PBP3 in the complex was shown in gray. Amino acid labels of the PBP3-ceftadizime complex and the apo structure are shown in black and orange, respectively. The stick model in cyan is ceftazidime. Black dashed lines indicate hydrogen bonds and salt bridges in the complex. Yellow dashed lines indicate a hydrogen bond and salt bridges in the apo structure. Data originate from PDB codes 3PBO and 3PBN.

In the apo structure, besides the above-described Asp525-Lys490-Glu526 interaction, Glu526 forms a hydrogen bond with the Tyr503 carbonyl backbone (Fig. 4). Tyr503 forms a van der Waals interaction with Thr487 in the KSGT motif. The Thr487 carbonyl backbone forms a hydrogen bond with the Tyr409 side chain. When ceftazidime binds to PBP3, the Arg504 side chain forms salt bridges with the Asp525 side chain (Fig. 4). The conformational change of Arg504 facilitates the structural arrangement of Tyr409, Thr487, Arg489, and Tyr503 and places ceftazidime in the active site pocket. In the active site pocket, Thr487 abrogates interactions with Tyr409 and Tyr503, forming hydrogen bonds with ceftazidime at the carboxylic acid, carbonyl, and amide groups in C4, C8, and C7 positions, respectively (Fig. 4), whereas Arg489 and Tyr409 stabilize the aminothiazole moiety of ceftazidime (16). Tyr503 forms an aromatic wall with Tyr532 and Phe533 to stabilize the dem-dimethyl group of ceftazidime (15). Furthermore, Arg504 forms van der Waals interactions with Phe290 and facilitates hydrogen bond formation between Glu291 and aminothiazole amine NH_2_ of ceftazidime (15, 22). Therefore, the replacement of Arg504 with cysteine might prevent the interaction of the residue with Asp525 and Phe290, consequently reducing the stabilization effect of Glu291, Tyr409, Thr487, Arg489, and Tyr503 on ceftazidime.

## DISCUSSION

PBP3 mutations represent one type of acquired mutation occurring during chemotherapy in patients with *P. aeruginosa* infection in recent years (9, 10, 12). To clarify the PBP3 mutations that alter β-lactam susceptibility, this study formally analyzed the effect of a single SNP found in a meropenem-resistant isolate on phenotype under a common genetic background. This study found positive fitness effects in broth microdilution method or growth curves for all seven tested PB domain mutations in the presence of carbapenems. However, unexpectedly, the seven mutations increased susceptibility to cefiderocol and piperacillin.

The seven PB domain positions considerably reduced the susceptibility of *P. aeruginosa* to doripenem and meropenem, but the effect on imipenem was minor. We speculate that this may be due to the lower binding affinity of imipenem for *P. aeruginosa* PBP3 than meropenem (23, 24). PBP3 variants containing the seven PB domain mutations can be considered carbapenem-adapted forms. Although no formal experimental evidence is available, we speculate that other rare mutations near C-terminal PB domain positions (Val465 to Ser538) will have a similar effect on carbapenem susceptibility. As the most common PB domain mutation, Arg504Cys reduced susceptibility to both cephems (ceftazidime, cefepime, and ceftolozane-tazobactam) and carbapenems (meropenem, doripenem, and imipenem). The Arg504Cys mutation was indeed identified as an acquired mutation in a *P. aeruginosa* clone that evolved in the presence of ceftazidime *in vitro* (12) and a patient (25). Furthermore, Shields et al. (10) isolated *P. aeruginosa* clones carrying the Met460Thr or Arg504Cys mutation from patients exposed to ceftolozane-tazobactam. Notably, the Arg504Cys mutation alone reduced the bacterium’s susceptibility to ceftolozane-tazobactam but the Met460Thr mutation alone did not. Although the effect was minor compared with that of Arg504Cys, the positive effect of Met460Thr, Val537Leu, Pro527Ser, and Pro527Thr on growth with cefepime was significant in growth parameters. Therefore, besides Arg504Cys, these mutations can also be recognized as a signal for adaptation to cephems. Shields et al. (10) cited the Met460Thr and Arg504Cys mutations as factors associated with reduced cefiderocol susceptibility. However, our observation suggests that the reduced cefiderocol susceptibility in their isolates is attributable to other co-occurring mutations in AmpC or PirR but not to the PB domain mutations in FtsI/PBP3.

Glen and Lamont (26) recently characterized five FtsI/PBP3 mutations (Arg504Cys, Val471Gly, Phe533Leu, Val537Leu, and Ala244Leu) in the PAO1 wild-type chromosome background using a different set of antimicrobials and the agar dilution method with 24 h incubation time routine. Their conclusions on the nature of Arg504Cys, Val471Gly, Phe533Leu, and Val537Leu overlap with our conclusion. However, the aztreonam MIC was increased by Arg504Cys and Val537Leu and that of cefepime was increased by Val537Leu in their study. Imipenem MIC did not increase in their study. This inconsistency may stem from several factors in addition to the difference in the genetic background of the strain used. The effect of PB domain mutations was evidenced in growth parameters in surprisingly different ways among antimicrobials. Notably, we detected the positive effect of PB domain mutations on growth in the presence of imipenem as a shortened lag phase but no difference in the maximum OD or maximum slope. Thus, the lack of detectable effects of PB domain mutations on the imipenem MIC in the previous study could partially be attributable to the difference in the incubation duration between our study and the previous study. Although the effects of Arg504Cys and Val537Leu on aztreonam MIC were not detected in our susceptibility test, their positive effects on growth were detected as increases in the maximum OD and AUC, but not in the maximum slope in growth curves (Fig. 2). Thus, differences in the incubation duration may explain the differences in the aztreonam MIC patterns between studies. The positive fitness effects of Val537Leu and Met460Thr in the presence of cefepime were also detected as increases in growth parameters but not in MIC in our test. Therefore, the inconsistency in the MIC patterns of antibiotics among studies can be attributed to the limitations of susceptibility tests that use serial twofold dilutions of antibiotics or limitations in using cell density, but not CFUs as a measure of bacterial growth in the broth microdilution method. Finally, we used the *mex* operon deletion mutant as a model strain. Compared with the parent strain PAO1, our model strain YM64 exhibited a lower maximum slope in the exponential phase (Fig. S3). Therefore, the incubation time required to reach visually detectable turbidity can generally be longer for YM64 than for PAO1. Consequently, the mutational effects that influence only the lag phase length might be difficult to detect for PAO1 in short-term endpoint assays, whereas those of YM64 may be relatively easier. In addition to the characterization of Met460Thr and Pro527Ser, this study provided additional novel insights into the nature of the other five common PBP3 mutations in the presence of cefiderocol, doripenem, ceftolozane-tazobactam, and imipenem–relebactam, as well as growth parameters in the presence of representative β-lactams, thereby complementing the previous study.

Cocrystal structures of wild-type PBP3 with β-lactams suggest that, excluding Phe533, resistance-conferring mutations in the PB domain occurred at residues that do not directly interact with substrates. This might be because mutations of residues directly interacting with substrates affect reactions against natural substrates, harming bacterial fitness. All residues in the mutation positions can affect the mobility of Tyr409, Arg489, Try503, Tyr532, Phe533, or the KSGT motif upon substrate binding via intramolecular interactions. Tyr409 and Arg489 are key residues for stabilizing the aminothiazole moiety of ceftazidime (16). Try503, Tyr532, and Phe533 were key residues of aromatic wall formation for ceftazidime binding, whereas Tyr532 and Phe533 were key residues of hydrophobic pillar formation for meropenem binding (16). Doripenem and meropenem have a common structure consisting of a carbapenem core, hydroxyethyl group at the C6 position, and a pyrrolidine-linked sulfur at the C2 position. Thus, most of the aforementioned PBP3-meropenem interactions should also apply to PBP3-doripenem interactions. Arg504 can affect the mobility of Glu291, Tyr409, and Arg489, which directly interacts with the aminothiazole moiety of ceftazidime. Cefepime also has an aminothiazole group. Thus, the influence of the Arg504Cys mutation on the interactions between the three residues and the substrate should apply to PBP3-cefepime interactions.

Mechanisms by which PB domain mutants increase the susceptibility to cefiderocol and piperacillin are unclear. Cefiderocol has an aminothiazole group in the C7 side chain ceftadizime; however, cefiderocol has a chlorocatechol group in the C3 side chain. The interactions of PBP3 with the C3 side chain of cefiderocol may differ between wild-type PBP3 and PB domain mutants.

PBP3 catalyzes the hydrolysis of piperacillin into (*5R*)-penicilloic acid, which is further converted into (*5S*)-penicilloic acid (27). Therefore, the increase in piperacillin susceptibility may be associated with the changes in the interactions with the product as well as the substrate piperacillin. Currently, the amino acid interactions and relevant reaction steps are difficult to pinpoint without determining a complex structure for specific PBP3 variants carrying a mutation, such as those in Val471Gly, Phe533Leu, and Phe537Leu, which have a considerable impact on the susceptibility to piperacillin.

In countries with a low carbapenemase prevalence such as Japan (14) and the USA (13), *P. aeruginosa* should acquire chromosomal resistance mutations. This study reinforced that in addition to inactivated *oprD*, PB domain mutations in PBP3 are hallmarks of meropenem/doripenem resistance and ceftazidime/cefepime resistance. Although genomic data regarding *P. aeruginosa* are rapidly being accumulated, the link between chromosomal variant alleles and the resistance phenotype has not been sufficiently examined. Continuous efforts are needed to study the nature of variant alleles in CR-PA genomes to build a foundation for antimicrobial stewardship based on clinical sequencing.

This study has a limitation. This study did not analyze the combinatorial effects of co-occurring mutations and efflux systems on the susceptibility to β-lactams. This point should be clarified in future research.

In conclusion, seven specific PB domain mutations found in CR-PA PBP3 considerably reduce the bacterium’s susceptibility to carbapenems carrying pyrrolidine-linked sulfur at the C2 position. Cefiderocol would have efficacy against the evolved clinical isolates possessing the seven PB domain mutations.

## MATERIALS AND METHODS

### Strains, plasmids, and culture media

The strains and plasmids used in this study are listed in Table 3. Our model *P. aeruginosa* strain YM64, which lacks RND family efflux pumps (20), showed a lower maximum growth rate and turbidity than the parent strain PAO1 in batch culture (Fig. S3). *P. aeruginosa* and *E. coli* strains were cultured in BD Difco Luria-Bertani (LB) broth Lennox (BD Biosciences, Franklin Lakes, NJ, USA). Cation-adjusted MHB (BD Biosciences) was used for antimicrobial susceptibility testing and growth parameter analysis for *P. aeruginosa* strains. When required, tetracycline (Tc) was added to the medium at 2.5 µg/mL. Iron-depleted cation-adjusted MHB was prepared for growth assays using Chelex 100 resins (Bio-Rad Laboratories, Inc., Hercules, CA, USA) following CLSI M07 12th Edition (Clinical Laboratory Standards Institute). M9 minimal medium agar containing 30 mM succinate and Tc was used to select recombinant *P. aeruginosa* clones from a mating mixture. TYS agar medium (3 g tryptone, 1 g yeast extract, 15 g agar, and 50 g sucrose per 1 L) was used for counter-selection for *sacB* of pEX18Tc (28).

**TABLE 3:** Strains and plasmids used

Strains or plasmids	Genotype and relevant characteristics	References
*P. aeruginosa*		
YM64	PAO1Δ(*mexAB-oprM*) Δ(*mexCD-oprJ*) Δ(*mexEF-oprN*) Δ*mexXY*	20
PAY7	YM64 *ftsI* (C265G); FtsI (Leu89Val)	This study
PAY18	YM64 *ftsI* (C1579A); FtsI (Pro527Thr)	This study
PAY53	YM64 *ftsI* (T1379C); FtsI (Met460Thr)	This study
PAY49	YM64 *ftsI* (T1412G); FtsI (Val471Gly)	This study
PAY42	YM64 *ftsI* (C1599A); FtsI (Phe533Leu)	This study
PAY45	YM64 *ftsI* (C1579T); FtsI (Pro527Ser)	This study
PAY47	YM64 *ftsI* (C1510T); FtsI (Arg504Cys)	This study
PAY51	YM64 *ftsI* (G1609C); FtsI (Val537Leu)	This study
*E. coli*		
DH5α	F^−^, Φ80d*lacZ*ΔM15, Δ(*lacZYA-argF*)U169, *deoR*, *recA*1, *endA*1, *hsdR*17(r_K_^−^, m_K_^+^), *phoA*, *supE*44, λ^−^, *thi*-1, *gyrA*96, *relA*1	Nippon gene Inc.
S17-1	F^−^, *thi*, *pro*, *hsdR*, [RP4-2 *tetA*::Mu *aphA*::Tn*7*]; Tp^r^ Sm^r^	29
Plasmids		
pEX18Tc	Allelic exchange vector; Tc^r^; pUC*oriV*, *tetM*, *sacB*, *oriT*	28
pHY1631	pEX18Tc derivative carrying 4.4 kb segment containing *ftsI* (locus tag PA4418) of YM64 chromosome; Tc^r^	This study
pHY1632	pHY1631 derivative containing C265G substitution in *ftsI*; Tc^r^; FtsI (Leu89Val)	This study
pHY1633	pHY1631 derivative containing C1579A substitution in *ftsI*; Tc^r^; FtsI (Pro527Thr)	This study
pHY1634	pHY1631 derivative containing C1599A substitution in *ftsI*; Tc^r^, FtsI (Phe533Leu)	This study
pHY1639	pHY1631 derivative containing C1510T substitution in *ftsI*; Tc^r^, FtsI (Arg504Cys)	This study
pHY1640	pHY1631 derivative containing C1579T substitution in *ftsI*; Tc^r^, FtsI (Pro527Ser)	This study
pHY1641	pHY1631 derivative containing C1379T substitution in *ftsI*; Tc^r^; FtsI (Met460Thr)	This study
pHY1642	pHY1631 derivative containing G1609C substitution in *ftsI*; Tc^r^, FtsI (Val537Leu)	This study
pHY1643	pHY1631 derivative containing T1412G substitution in *ftsI*; Tc^r^, FtsI (Val471Gly)	This study

### Genome data set

The genomic sequences of meropenem-resistant *P. aeruginosa* strains were obtained by assembling publicly available reads (NCBI BioProject PRJNA824880) (13) and using our previously released genome assemblies associated with Japan Antimicrobial Resistant Bacterial Surveillance (14). In total, 1,354 draft genome assemblies were constructed using shovill v.1.1.0 (https://github.com/tseemann/shovill) and further filtered into 1,297 assemblies fulfilling L90 <200 and pairwise Mash distances of ≥1 × 10^−5^ to <0.06 (30) using PanACoTA v.1.4.1 (31) with the “prepare --l90 200 min_dist 1e-5” option. A phylogenetic tree of CR-PA was generated using their core gene alignment in the PanACoTA pipeline with the “pangenome -i 0.8” and “coreper -t 0.95” options and the GTR model of IQtree2 (32). The tree was visualized using the R packages phylotools, ggtree, and ggtreeExtra (https://www.r-project.org) (33, 34). The phylogenetic tree was rooted by the midpoint rooting method.

The *ftsI* sequences of the 1,297 strains were identified via tBLASTn searches (on BLAST v2.14.0+) (35) against an in-house generated nucleotide BLAST database using the FtsI/PBP3 sequence of strain PAO1 (RefSeq protein ID: NP_253108.1) as a query. The FtsI/PBP3 variants characterized in this study correspond to the following NCBI RefSeq protein IDs: Arg504Cys, WP_012614521.1; Val471Gly, WP_034022480.1; Pro527Ser, WP_014603572.1; Pro527Thr, WP_070142976.1; F533L, WP_033963613.1; Met460Thr, WP_210774036.1; and Leu89Val, WP_124170169.1.

### Molecular genetics experiments

The 4.4 kb *ftsI*-containing chromosomal segment of the *P. aeruginosa* PAO1 derivative strain YM64 (20) was PCR-amplified using the primers pEX-MCS-PA-ftsI-F and pEX-MCS-PA-ftsI-R (Table S2) and cloned into *Sma*I-digested pEX18Tc (28) using NEBuilder (New England Biolabs, Ipswich, MA, USA) and ECOS Competent *E. coli* DH5α (Nippon Gene Co., Ltd., Tokyo Japan). The resulting plasmid was named pHY1631. Nonsynonymous substitutions were introduced into the *ftsI* locus on pHY1631 in three different ways. In the first method, the entire pHY1631 plasmid was PCR-amplified using primers containing a substitution and KOD One polymerase (Toyobo, Shiga, Japan), and the products were phosphorylated, ligated, and transformed into *E. coli* DH5α. This was performed to introduce a C265G or C1579A substitution into pHY1631, yielding pHY1632 and pHY1633, respectively. A similar method was used to introduce a C1599A substitution into pHY1631, yielding pHY1634. However, 5′ phosphorylated primers were used, and the phosphorylation step was omitted. In the final method, half portions of pHY1631 were PCR-amplified using tet-ORF-F or tet-ORF-R (Table S2) and primer annealing to the substitution site. Then, the two PCR products were combined using NEBuilder. This method was used to introduce T1379C, T1412G, C1510T, C1579T, or G1609C substitution into pHY1631, yielding pHY1641, pHY1634, pHY1640, pHY1639, and pHY1642, respectively (Table 2).

pHY1631 derivatives carrying *ftsI* substitutions were introduced into the *E. coli* strain S17-1 (29), and the transformants were mated with the *P. aeruginosa* strain YM64 on LB agar overnight. The recombinant *P. aeruginosa* clones were screened on M9 succinate agar containing 2.5 µg/mL Tc. The candidate Tc^r^ YM64 recombinant clones were further replicated on LB agar containing Tc, and the colonies were further streaked on TYS agar to select for clones that lost the integrated vector. The colonies grown on TYS agar were replicated on LB agar and LB agar containing Tc. Then, 10–24 Tc-susceptible colonies were analyzed for the presence of the nonsynonymous substitution in *ftsI* by colony PCR with the primers FtsI-seq-F and FtsI-seq-R (Table S2), followed by Sanger sequencing of the PCR products using the same primers. Recombinant clones showing a single fluorescence signal from the introduced substitution at the expected position in the chromatogram file were stocked and selected for further phenotype analysis.

### Antimicrobial susceptibility testing

To determine β-lactam MICs, we used the microdilution method using MHB following Clinical and Laboratory Standards Institute guidelines. In brief, freshly prepared *P. aeruginosa* colonies were diluted in saline to MacFarland 0.5, further diluted, and inoculated into microtiter plates containing MHB and antibiotics. After inoculation, microtiter plates were incubated at 35°C. The MIC was determined after 20 h. We prepared 11 dilutions ranging from 0.0625 to 128 µg/mL for meropenem, doripenem, and imipenem; from 0.0313 to 32 µg/mL for piperacillin; from 0.0078 to 8 µg/mL for aztreonam; and from 0.0156 to 16 µg/mL for ceftazidime and cefepime. The cefiderocol MIC was determined using a dry plate containing iron-depleted medium and cefiderocol (concentration range 0.00325 to 32 µg/mL) prepared by Eeiken Chemical Co., Ltd. (Tokyo, Japan). Ceftolozane-tazobactam MIC and imipenem-relebactam MIC were determined using a frozen plate prepared by Eeiken Chemical Co., Ltd. (concentration range from 0.025 to 64 µg/mL for ceftolozane or imipenem and 4 µg/mL for tazobactam or relebactam).

### Growth parameter analysis

Five colonies of test strains were individually inoculated into 2 mL of MHB and cultured for 20 h with agitation at 35°C. The precultures were diluted by 500-fold in fresh cation-adjusted MHB or iron-depleted cation-adjusted MHB, and 100 µL of the diluted culture was inoculated into a 96-well microtiter plate. Antibiotics were added to the medium at the MIC or lower concentrations, under which most of the test strains exhibited S-shaped growth curves that are appropriate for growth parameter analysis. Antibiotics were added to the medium at the following concentrations (μg/mL): meropenem, 0.125; imipenem, 0.5 or 1; piperacillin, 0.063; aztreonam, 0.125; cefepime, 0.063; and cefiderocol, 0.002. The plates were covered with Breathe-Easy sealing membrane (Merck, Burlington, MA, USA) and incubated without agitation for 24 h (48 h for cefiderocol) at 35°C in BioTek LogPhase 600 (Agilent Technologies Inc., Santa Clara, CA, USA). OD_600 nm_ was recorded every 10 min. The OD_600 nm_ data were analyzed using the R package grofit (36) to obtain growth parameters. In grofit, we used the *grofit.control* function with “interactive = F, smooth.gc = (0.4), model.type = ‘logistic,’ nboot.gc = 100” option. “mu.bt,” “A.bt,” and “integral.bt” values obtained by the bootstrap sampling method were considered as the maximum slope (shift in OD/h), maximum OD, and AUC, respectively, of the growth curves. Growth parameters were compared between strain YM64 carrying wild-type PBP3 and the recombinant strain carrying variant PBP3 by the two-sided Wilcoxon rank sum test, followed by multiple testing correction using the Benjamini-Hochberg method.

### Molecular graphics

Crystal structures of *P. aeruginosa* wild-type PBP3 were obtained from Protein Data Bank (https://www.rcsb.org). Superposition of the PBP3-meropenem complex (PDB code: 3PBR) or PBP3-ceftazidime complex (PDB code: 3PBO) onto the apo structure (PDB code: 3PBN) was performed using the SUPERPOSE program in CCP4 (37). Molecular graphic images were generated using PyMOL (Schrödinger, LLC, Cambridge, MD, USA).

## Data Availability

The phylogenetic tree, *ftsI* alignments, and R scripts used in this study are available in the figshare repository (DOI: 10.6084/m9.figshare.27281901) (38). The CR-PA draft genomes were obtained from previously published raw reads linked to BioProjects PRJNA824880 and PRJDB16993 (39, 40). The shovill assemblies are available in the Zenodo repository (DOI: 10.5281/zenodo.13968581 [41] and DOI: 10.5281/zenodo.10693593 [42]).

## SUPPLEMENTAL MATERIAL

Fig. S1 spectrum.01027-25-s0001.docx Growth curves of YM64 and its derivative strains producing PBP3 variants.

Fig. S2 spectrum.01027-25-s0002.docx Residues around Val537 in the PBP3-meropenem complex.

Fig. S3 spectrum.01027-25-s0003.docx Growth curves of YM64 and PAO1.

Table S1 spectrum.01027-25-s0004.xlsx Nonsynonymous substitutions detected in ftsI of 1,297 CR-PA genomes.

Table S2 spectrum.01027-25-s0005.docx Oligonucleotides used.

ASM does not own the copyrights to Supplemental Material that may be linked to, or accessed through, an article. The authors have granted ASM a non-exclusive, world-wide license to publish the Supplemental Material files. Please contact the corresponding author directly for reuse.
