# Supplementary material for: Specific variants in ftsI reduce carbapenem susceptibility in Pseudomonas aeruginosa
Source: Microbiol Spectr. 2025 Jul 7;13(8):e01027-25. doi: 10.1128/spectrum.01027-25 (PMC12323637; doi:10.1128/spectrum.01027-25)
Supplement: Fig. S1 — Growth curves of YM64 and its derivative strains producing PBP3 variants. [file spectrum.01027-25-s0001.docx]

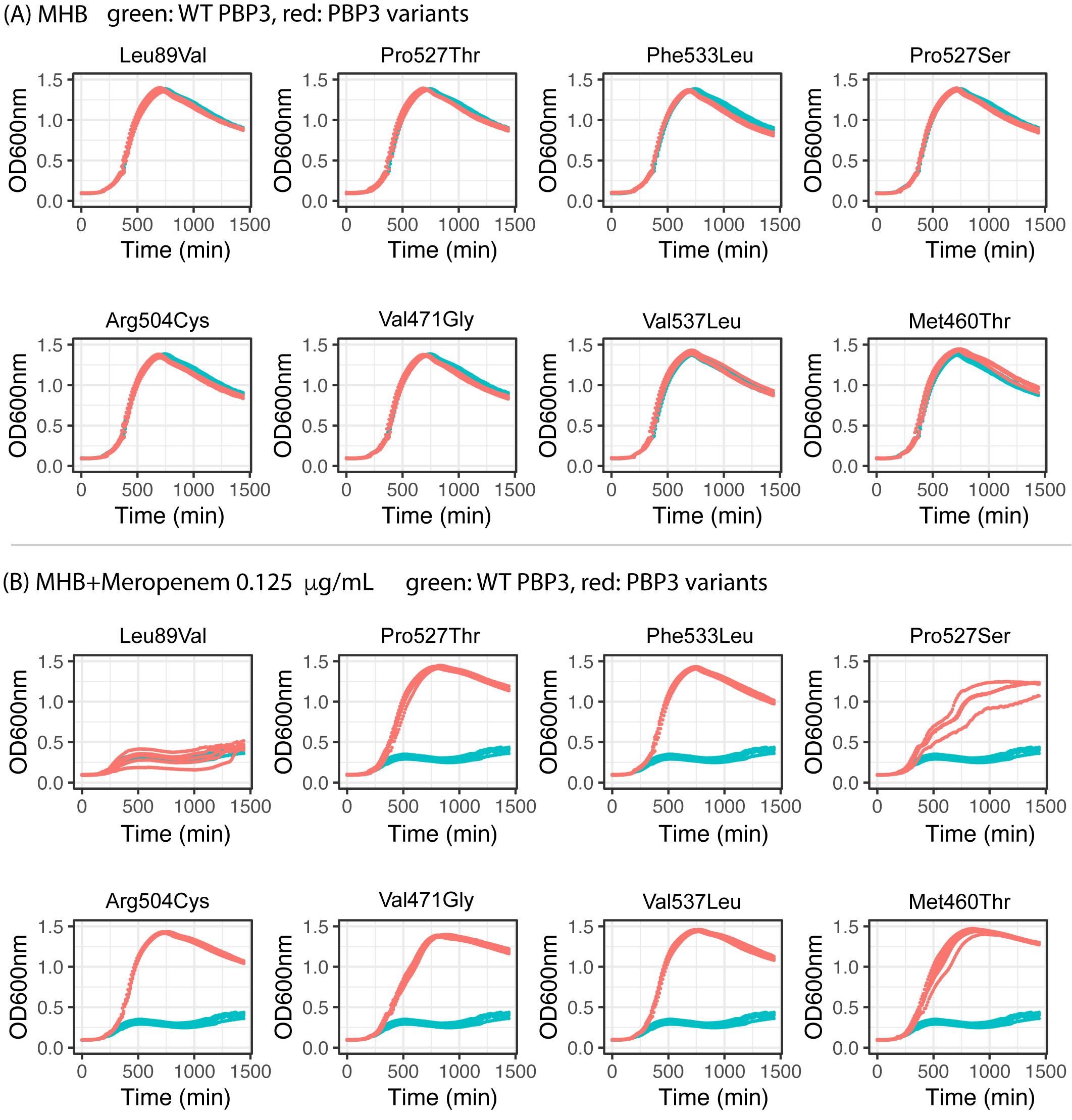


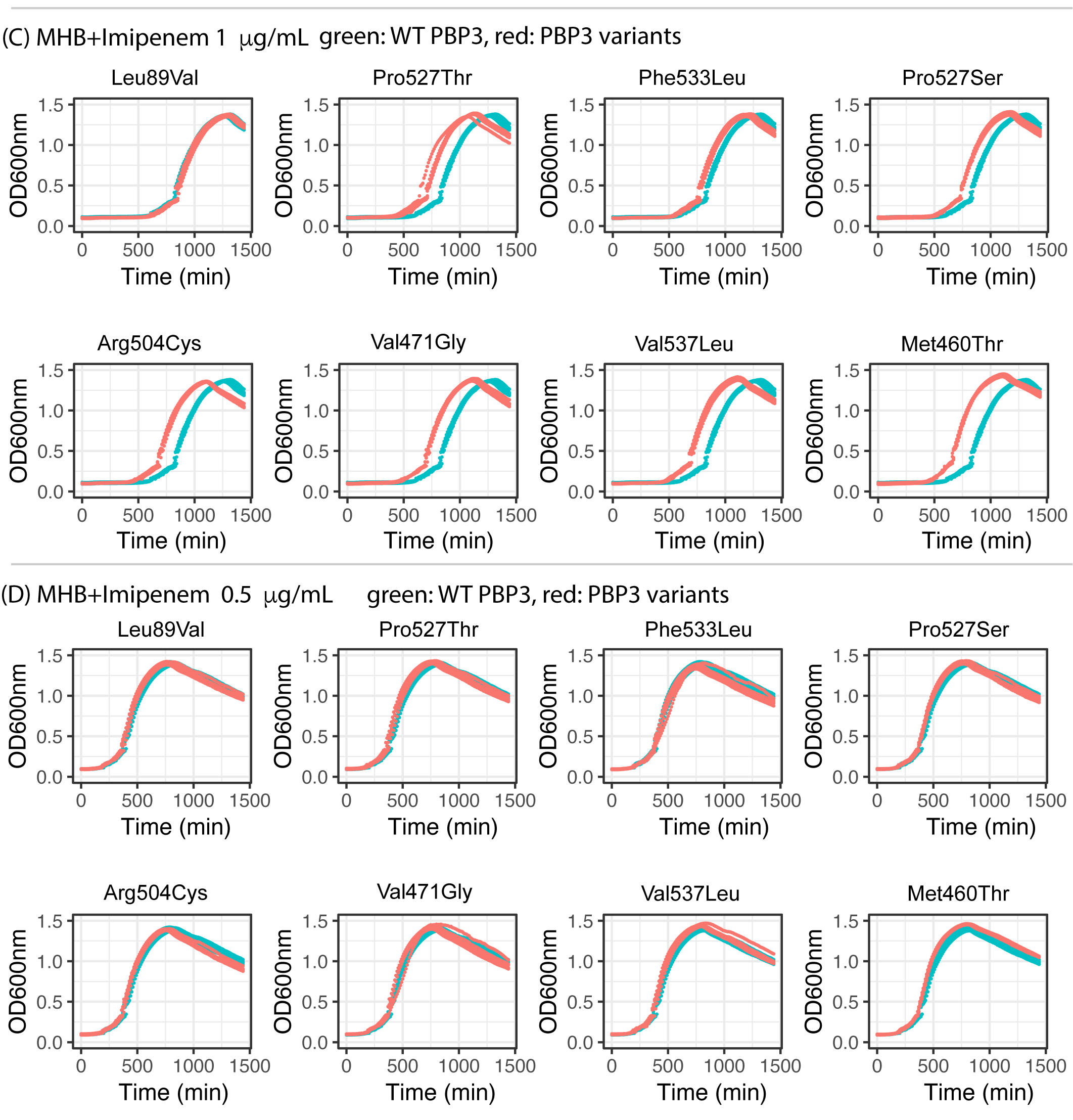


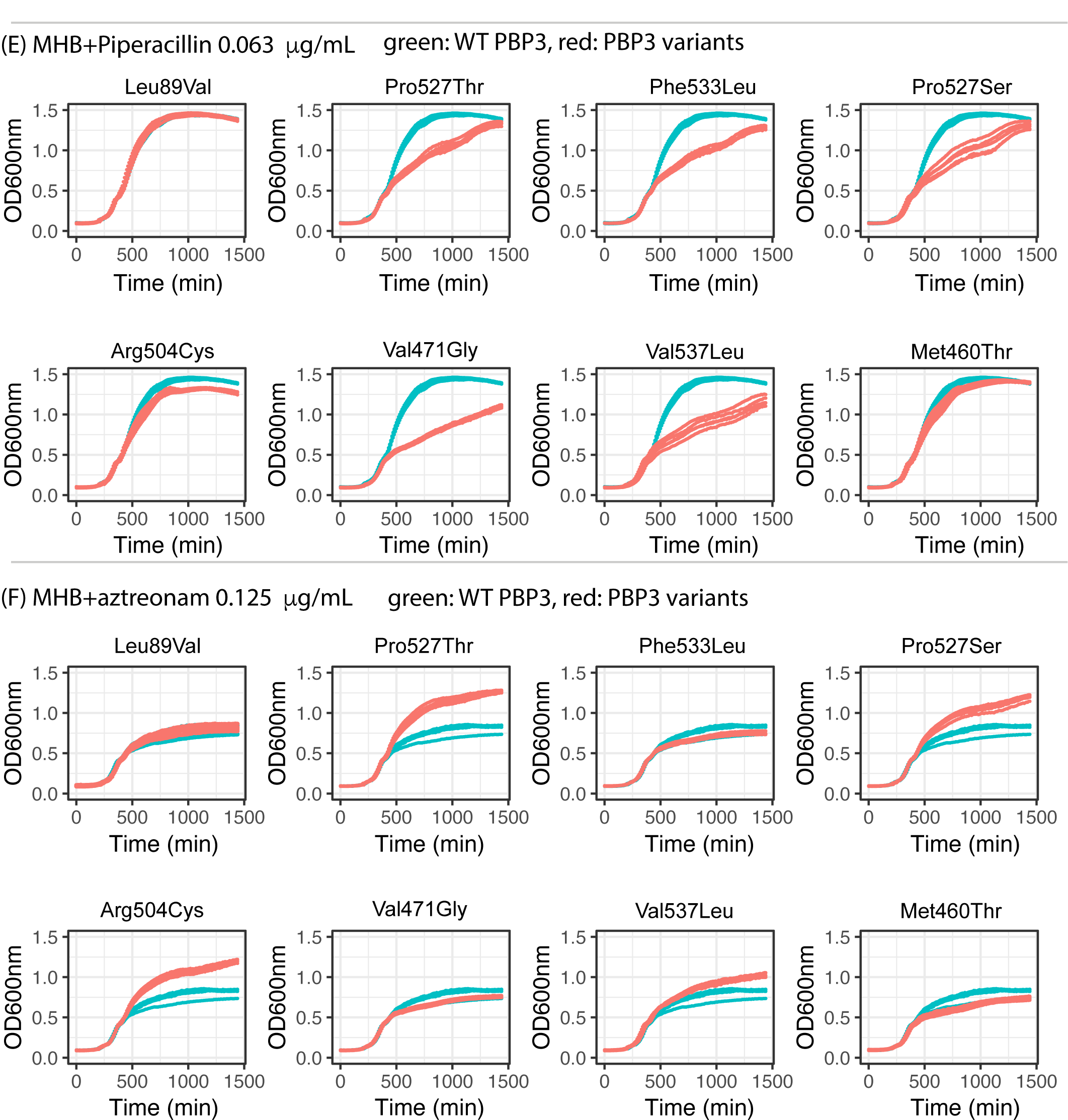


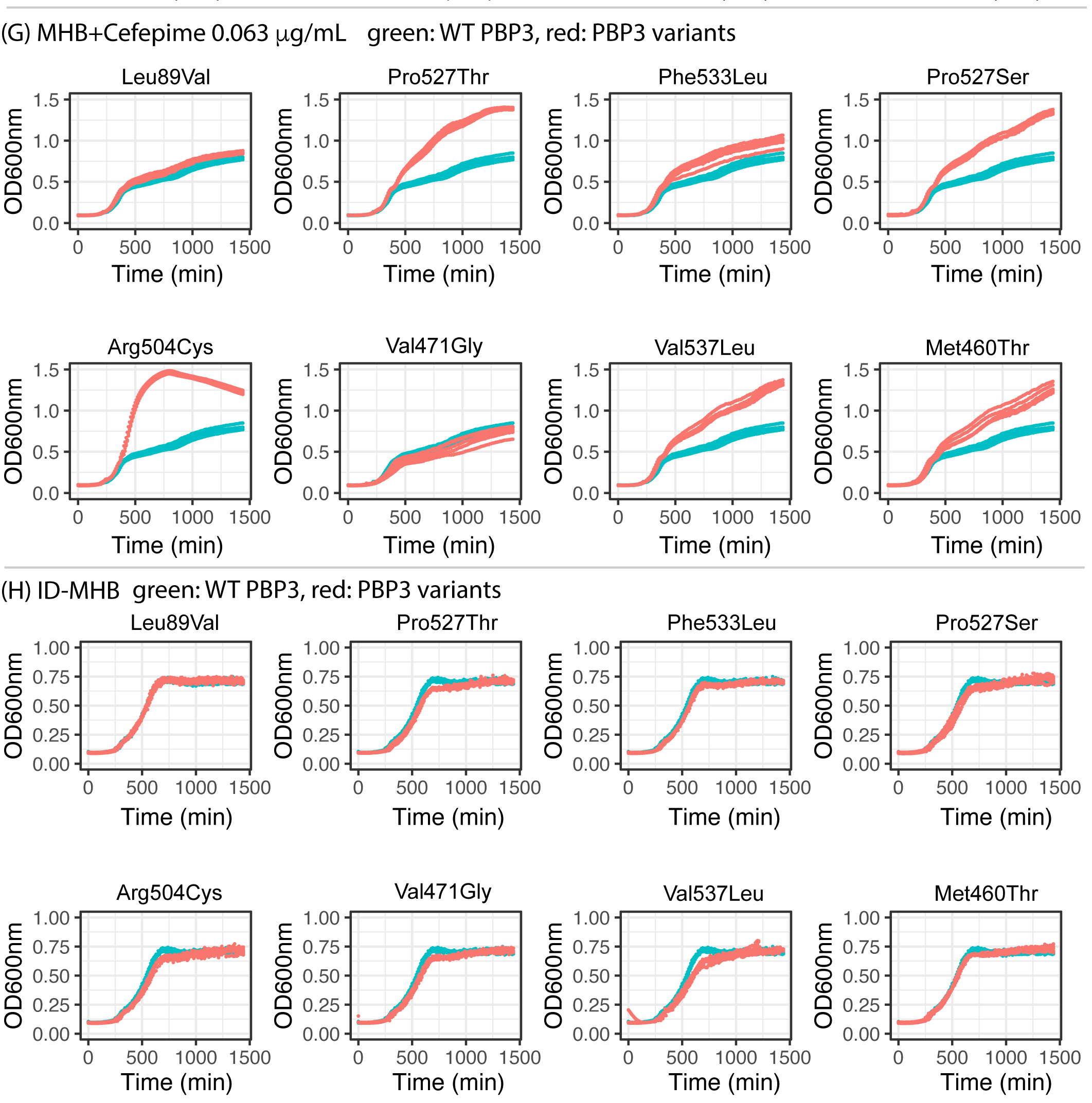


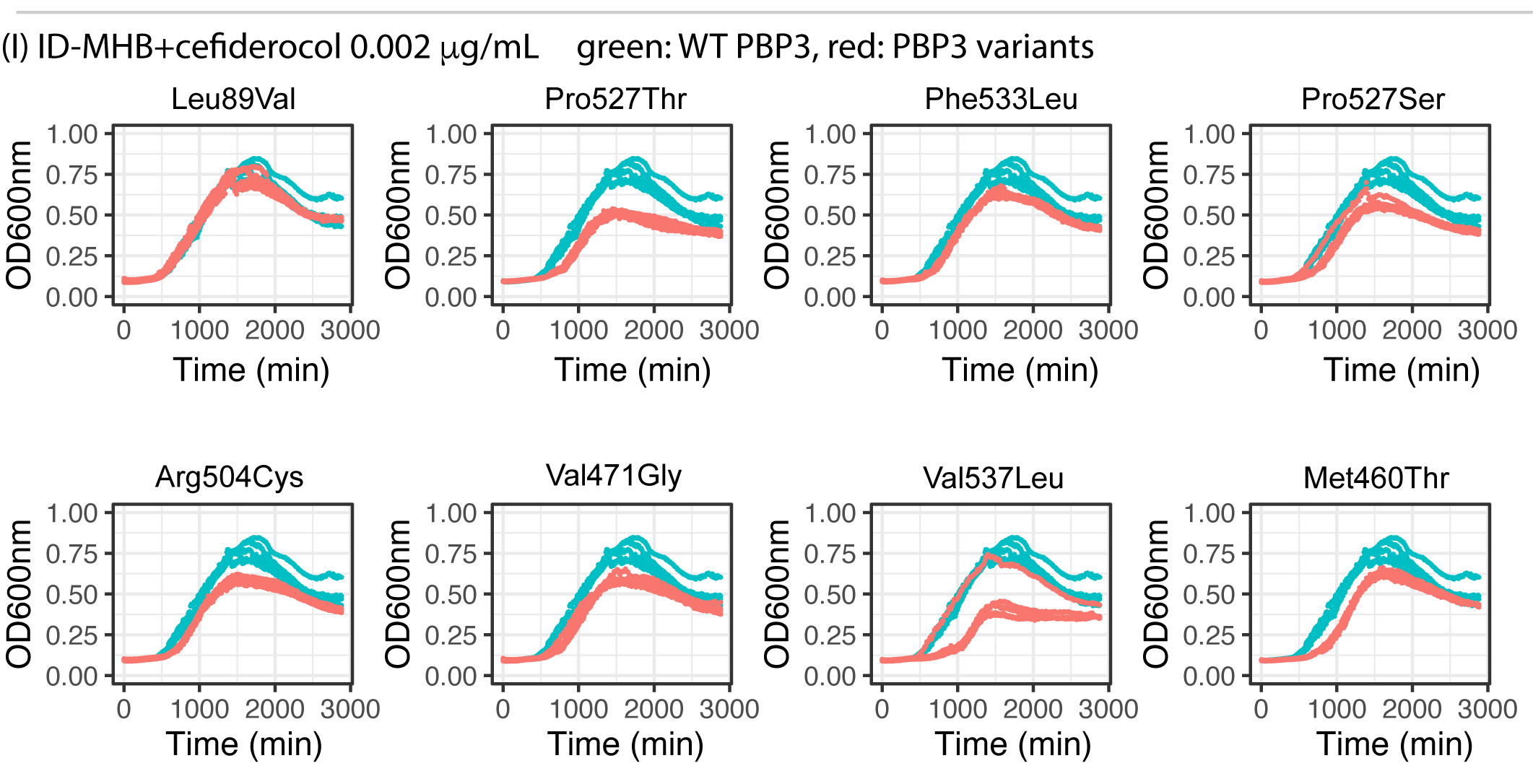


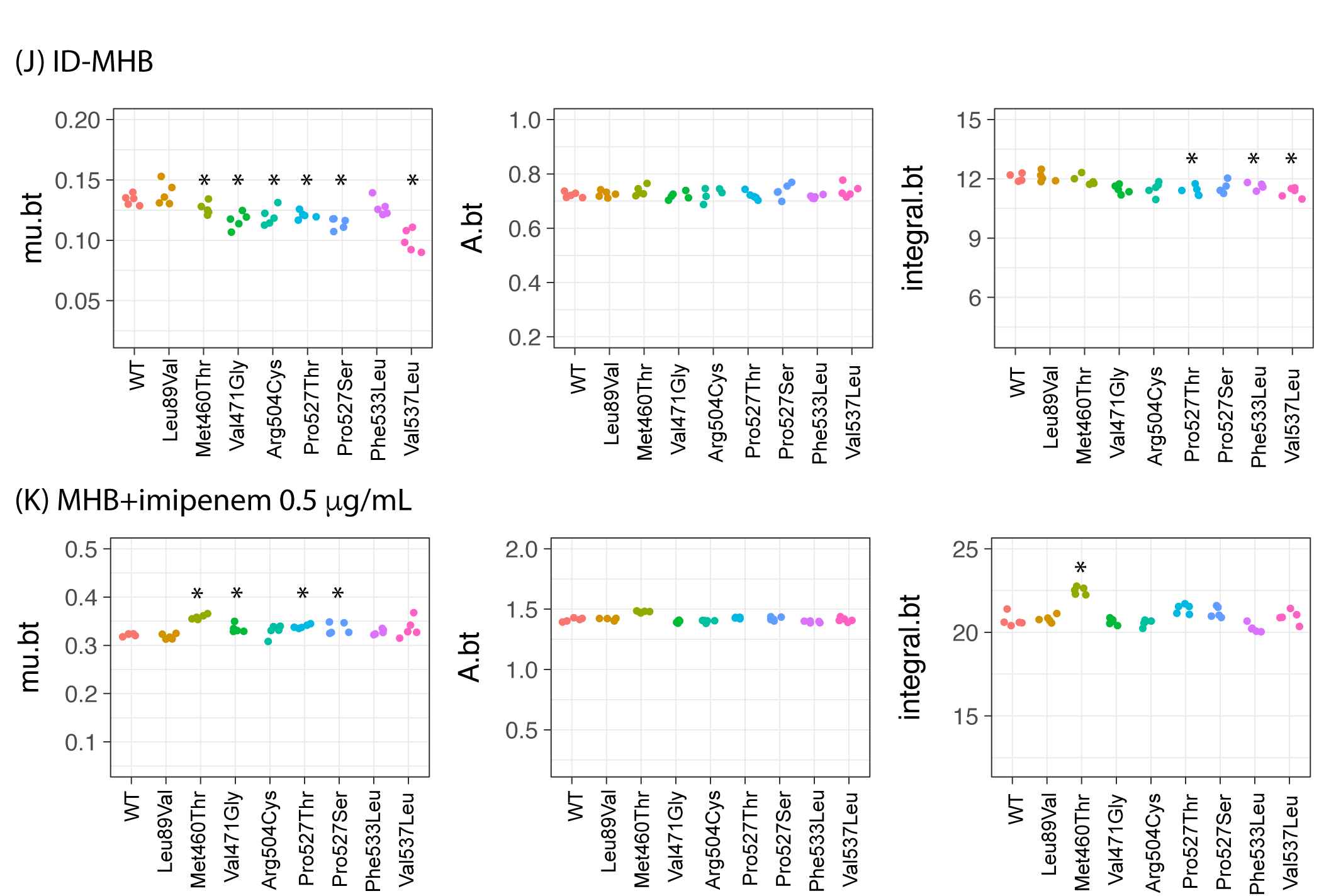


**Fig. S1.** Growth curves of strain YM64 and its derivative strains. (**A**) cation-adjusted Mueller-Hinton broth (MHB). Unit of the X-axis is minute. The OD values of five replicate cultures are shown in the same plot area. The OD data of YM64 harboring wild-type PBP3 is shown in green dots and that of strains harboring PBP3 mutations is show in red dots. (**B**) MHB containing meropenem at 0.125 μg/mL, (**C**) imipenem at 1μg/mL, (**D**) imipenem at 1 μg/mL, (**E**) piperacillin at 0.063 μg/mL, (**F**) aztreonam at 0.125 μg/mL, and (**G**) cefepime 0.063 μg/mL. (**H**) Iron-depleted MHB (ID-MHB). (**I**) Iron-depleted MHB containing cefiderocol at 0.002 μg/mL. (**J**) Growth parameters in iron-depleted MHB (ID-MHB). mu.bt: maximum slope. A.bt: maximum OD. Integral.bt: area under the curve. (**K**) Growth parameters in MHB containing imipenem at 0.5 μg/mL.
