# Supplementary material for: Specific variants in ftsI reduce carbapenem susceptibility in Pseudomonas aeruginosa
Source: Microbiol Spectr. 2025 Jul 7;13(8):e01027-25. doi: 10.1128/spectrum.01027-25 (PMC12323637; doi:10.1128/spectrum.01027-25)
Supplement: Fig. S2 — Residues around Val537 in the PBP3-meropenem complex. [file spectrum.01027-25-s0002.docx]

**
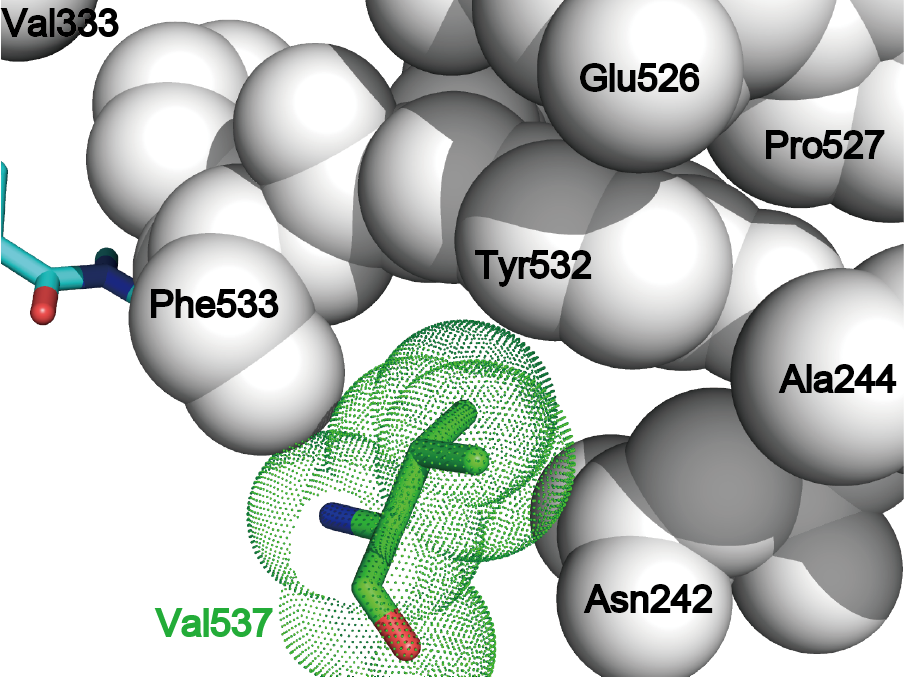
**

**Fig. S2**. Residues around Val537 in the PBP3–meropenem complex. Green sphere, Val537; gray sphere, residues other than Val537; cyan, meropenem.
