# Supplementary material for: Specific variants in ftsI reduce carbapenem susceptibility in Pseudomonas aeruginosa
Source: Microbiol Spectr. 2025 Jul 7;13(8):e01027-25. doi: 10.1128/spectrum.01027-25 (PMC12323637; doi:10.1128/spectrum.01027-25)
Supplement: Fig. S3 — Growth curves of YM64 and PAO1. [file spectrum.01027-25-s0003.docx]

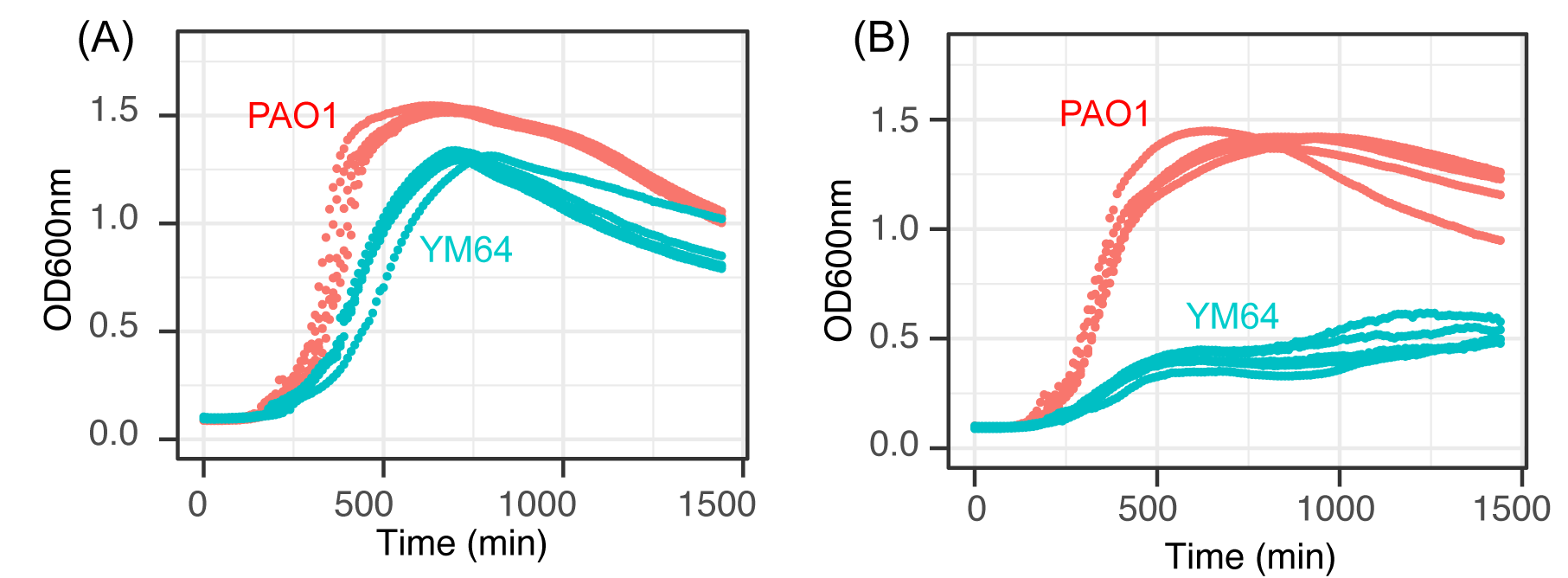


**Fig. S3.** Growth curves of strains YM64 and PAO1 in cation-adjusted Mueller-Hinton broth without antibiotics (**A**) and with meropenem (0.125 μg/ml) (**B**). Five replicate data were obtained from each strain and shown in the same color in the plot. Red: PAO1. Green, YM64. Maximum slopes of PAO1 and YM64 in the absence of antibiotics are 0.56±$0.07$ (mean±SD), 0.29$0.0$2, respectively. Maximum OD of PAO1 and YM64 are 1.53±0.01, and 1.32±0.02, respectively. Maximum slopes of PAO1 and YM64 in the presence of meropenem 0.125 μg/ml are 0.38±$0.05$ and 0.08 $0.0$1, respectively. Maximum OD of PAO1 and YM64 are 1.41±0.03 and 0.53±0.05, respectively.
