# Supplementary material for: Specific variants in ftsI reduce carbapenem susceptibility in Pseudomonas aeruginosa
Source: Microbiol Spectr. 2025 Jul 7;13(8):e01027-25. doi: 10.1128/spectrum.01027-25 (PMC12323637; doi:10.1128/spectrum.01027-25)
Supplement: Table S2 — Oligonucleotides used. [file spectrum.01027-25-s0005.docx]

**Table S2. Oligonucleotides used**

| Oligo name | Sequence (5´ to 3´)^a^ | Purpose |
| --- | --- | --- |
| pEX-MCS-PA-ftsI-F | TTCGAGCTCGGTACCCCCTGCGCGAGTACGCCAAGC | Cloning of *ftsI* region |
| pEX-MCS-PA-ftsI-R | ACTCTAGAGGATCCCCGCGAAGCCTTTGCGGATATCG | Cloning of *ftsI* region |
| ftsI_seq_F | ATGAAACTGAATTATTTCCAGGGCGC | Sequencing of *ftsI* |
| ftsI_seq_R | CTTTTGCGGGCGCAGCATTG | Sequencing of *ftsI* |
| P-c1599a-F | P-AGGCGGCCTGGTGTCGGCGC | Introduction of C1599A substitution into *ftsI* |
| P-c1599a-R | P-AAGTAGCCCGCCTTGCTCGG | Introduction of C1599A substitution into *ftsI* |
| L89V-c265g-F | GTGATGACCGCCAAGGAACGC | Introduction of C265G substitution into *ftsI* |
| L89V-c265g-R | CTCCTTGGGGTTGGCCCAC | Introduction of C265G substitution into *ftsI* |
| P527T-c1579a-F | ACGAGCAAGGCGGGCTACTTC | Introduction of C1579A substitution into *ftsI* |
| P527T-c1579a-R | CTCGTCGATCACCACGACCA | Introduction of C1579A substitution into *ftsI* |
| tet-ORF-F | GTCGGAATGGACGATATCCCGCAAGAGG | Introduction of SNP into *ftsI* on pHY1631 by recombination |
| tet-ORF-R | ATCGTCCATTCCGACAGCATCGCCAGTCAC | Introduction of SNP into *ftsI* on pHY1631 by recombination |
| R504C-c1510t-F | CGCCTATTGCTCGCTGTTCGCCGGTTTC | Introduction of C1510T substitution into *ftsI* by recombination |
| R504C-c1510t-R | AGCGAGCAATAGGCGTTTTCCCGGTAG | Introduction of C1510T substitution into *ftsI* by recombination |
| P527S-c1579t-F | CGACGAGTCGAGCAAGGCGGGCTAC | Introduction of C1579T substitution into *ftsI* by recombination |
| P527S-c1579t-R | TTGCTCGACTCGTCGATCACCACGAC | Introduction of C1579T into *ftsI* by recombination |
| M460T-t1379c-F | GCAGGGCACGCTGCAACAAGTGGTCG | Introduction of T1379C substitution into *ftsI* by recombination |
| M460T-t1379c-R | TTGCAGCGTGCCCTGCACGGTGGAAGC | Introduction of T1379C substitution into *ftsI* by recombination |
| V537L-g1609c-F | CGGCCTGCTGTCGGCGCCGGTGTTCAGTA | Introduction of G1609C substitution into *ftsI* by recombination |
| V537L-g1609c-R | TGAACAGCGGCGCCGACACCAG | Introduction of G1609C substitution into *ftsI* by recombination |
| V471G-t1412g-F | CGGGGGGTTCCGCGCCCAGGTGC | Introduction of T1412G into *ftsI* by recombination |
| V471G-t1412g-R | GCGCGGAACCCCCCGCCCTGGGCC | Introduction of T1412G into *ftsI* by recombination |

^a^ P indicates phosphorylation.
